# Demographic responses to climate‐driven variation in habitat quality across the annual cycle of a migratory bird species

**DOI:** 10.1002/ece3.8934

**Published:** 2022-06-11

**Authors:** James F. Saracco, Renée L. Cormier, Diana L. Humple, Sarah Stock, Ron Taylor, Rodney B. Siegel

**Affiliations:** ^1^ The Institute for Bird Populations Petaluma California USA; ^2^ 7620 Point Blue Conservation Science Petaluma California USA; ^3^ 233212 Division of Resources Management and Science Yosemite National Park El Portal California USA

**Keywords:** avian demography, black‐headed grosbeak, California, climate variation, integrated population model, MAPS program, Mexico, migratory connectivity

## Abstract

The demography and dynamics of migratory bird populations depend on patterns of movement and habitat quality across the annual cycle. We leveraged archival GPS‐tagging data, climate data, remote‐sensed vegetation data, and bird‐banding data to better understand the dynamics of black‐headed grosbeak (*Pheucticus melanocephalus*) populations in two breeding regions, the coast and Central Valley of California (Coastal California) and the Sierra Nevada mountain range (Sierra Nevada), over 28 years (1992–2019). Drought conditions across the annual cycle and rainfall timing on the molting grounds influenced seasonal habitat characteristics, including vegetation greenness and phenology (maturity dates). We developed a novel integrated population model with population state informed by adult capture data, recruitment rates informed by age‐specific capture data and climate covariates, and survival rates informed by adult capture–mark–recapture data and climate covariates. Population size was relatively variable among years for Coastal California, where numbers of recruits and survivors were positively correlated, and years of population increase were largely driven by recruitment. In the Sierra Nevada, population size was more consistent and showed stronger evidence of population regulation (numbers of recruits and survivors negatively correlated). Neither region showed evidence of long‐term population trend. We found only weak support for most climate–demographic rate relationships. However, recruitment rates for the Coastal California region were higher when rainfall was relatively early on the molting grounds and when wintering grounds were relatively cool and wet. We suggest that our approach of integrating movement, climate, and demographic data within a novel modeling framework can provide a useful method for better understanding the dynamics of broadly distributed migratory species.

## Graphical abstract

GPS‐tagging data show distinct molting and wintering regions for populations of a migratory bird species black‐headed grosbeak (*Pheucticus melanocephalus*) that breed in California, USA. Drought conditions across the annual cycle and rainfall timing during the molting period are reflected in annual variation in habitat characteristics. Integrated population models that incorporate climate covariates from distinct periods of the annual cycle as drivers of demographic rates can inform variation and trends of populations at regional scales.

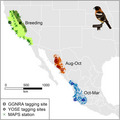

## 1 INTRODUCTION

The dynamics and trends of migratory animal populations depend on environments encountered across the annual cycle (Hostetler et al., 2015). For this reason, identifying networks of migratory connections represents a critical first step to the conservation of these species (Ruegg et al., 2020). Rapid advances in tracking technology have allowed for identification of these connections for a growing list of migratory animal species (McKinnon et al., 2013; Ruegg et al., 2014; Rushing et al., 2014). However, the value of this information for conservation is relatively limited without also identifying key environmental stressors at different points of the life cycle to better understand the consequences of migratory connections for population dynamics (Mancuso et al., 2021; Rushing, Ryder, & Marra 2016; Saracco & Rubenstein, 2020; Wilson et al., 2018).

**FIGURE 1:**
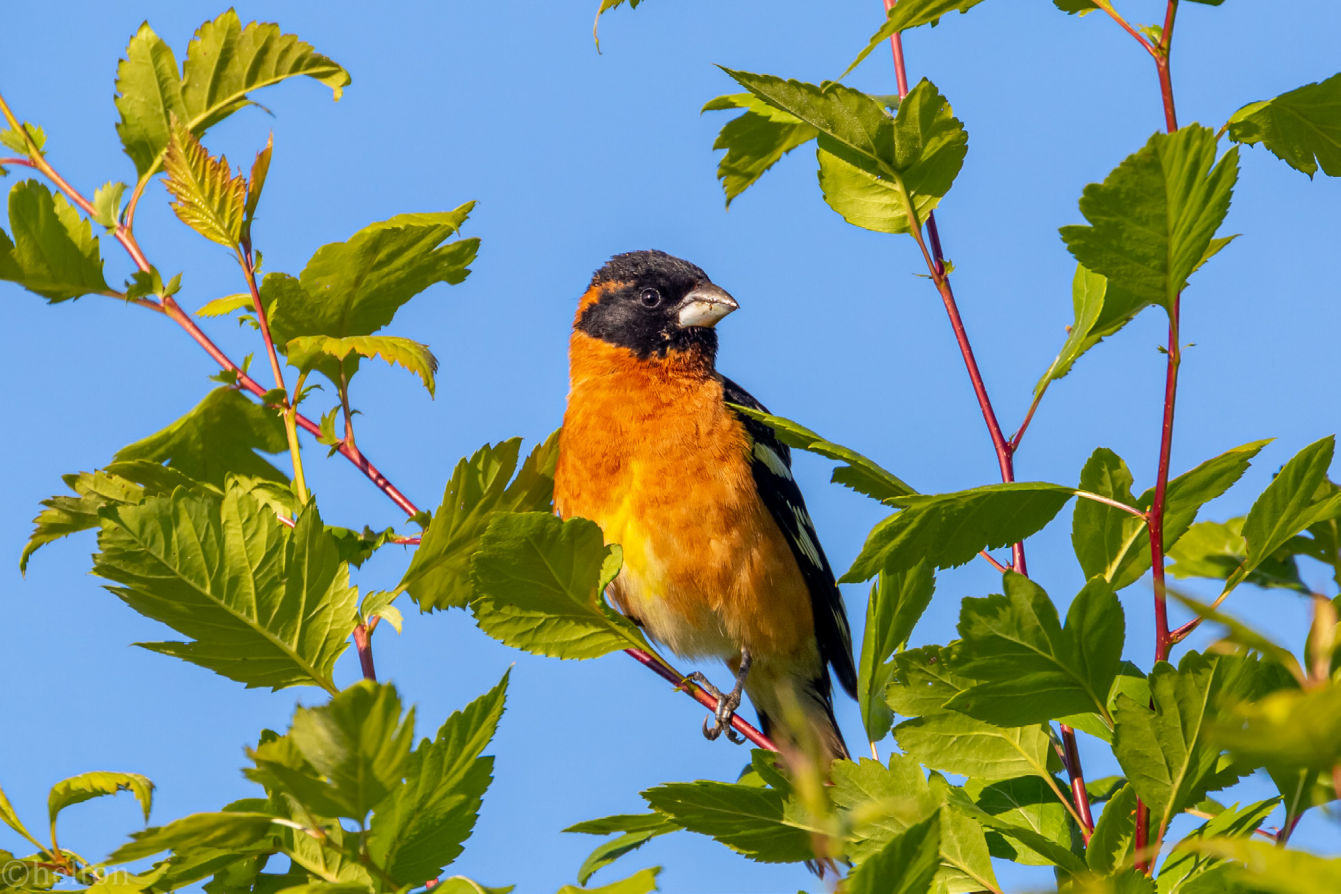
Black‐headed grosbeak (*Pheucticus melanocephalus*) is a migratory bird species that breeds in a variety of forested habitats of western North America, molts in the southwestern United States and northwestern Mexico, and overwinters in western Mexico (photo by C. Helton)

Studies aimed at parsing the relative importance of demographic and environmental drivers of population change across the annual cycle have been based on a model of two sedentary life history stages (breeding vs. non‐breeding) separated by brief spring and fall migratory periods (Rushing et al., 2017; Saracco & Rubenstein, 2020; Woodworth et al., 2017). However, additional periods of movement during the annual cycle of migratory birds may be more common than previously recognized (Pyle et al., 2018; Ruiz‐Gutierrez et al., 2016), and these complexities should also be considered in full annual cycle models (Pageau et al., 2020; Pyle et al., 2018). For example, black‐headed grosbeak (*Pheucticus melanocephalus*; Figure 1) is 1 of at least 19 bird species that breeds in seasonally arid regions of the western United States and has been documented migrating to the North American monsoon region (southwestern United States and northwestern Mexico) in late summer to undertake its annual prebasic molt prior to proceeding to overwintering areas farther south (Pageau et al., 2020; Pyle et al., 2009; Rohwer et al., 2005; Siegel et al., 2016).

Black‐headed grosbeak populations have increased across much of their breeding range over the past 50 years; however, populations in California and other parts of the southwestern United States have tended to decline (Sauer et al., 2020). We suggest that spatial and temporal variation in black‐headed grosbeak population dynamics and trends may reflect climate‐driven patterns of vegetation phenology and productivity at breeding, molting, and wintering sites. Identifying effects of climate variation on black‐headed grosbeak populations and their habitats will be critical for the conservation of this and other species that utilize seasonally arid habitats across western North America given observed and predicted climatic shifts due to climate change. Some observed and predicted effects of climate change in areas used by this species across the annual cycle include severe drought and reduced vegetation productivity on breeding grounds (Diffenbaugh et al., 2015; Goulden & Bales, 2019; Trujillo et al., 2012; Williams et al., 2020), changes in the timing and amount of rainfall on the molting grounds (Cook & Seager, 2013; Grantz et al., 2007; Méndez‐Barroso et al., 2009; Pascale et al., 2017), and warmer drier conditions on the wintering grounds (Karmalkar et al., 2011; Neelin et al., 2006).

Here, we develop an integrated population model to link 28 years (1992–2019) of data from black‐headed grosbeaks from a network of bird‐banding stations operated during the breeding season (Monitoring Avian Productivity and Survivorship [MAPS]; DeSante et al., 2004) to climate covariates from across the annual cycle (Wang et al., 2016), with a goal of assessing potential environmental drivers of vital rates and population dynamics in two breeding regions, the Sierra Nevada mountain range of California (hereafter Sierra Nevada) and along the coast and Central Valley of (hereafter Coastal California). The IPM framework allowed us to estimate and model recruitment rates using age‐specific capture data from adult birds, and adult survival rates based on adult capture–mark–recapture data for both regions. We assessed effects of the following climate covariates on grosbeak adult survival and recruitment: (1) drought on breeding grounds, (2, 3) drought and rainfall timing on molting grounds, and (4) drought on wintering grounds. We delineated molting and wintering grounds based on locations and habitat relationships of four GPS‐tagged birds that were captured and subsequently recaptured at MAPS stations. Finally, to better understand links between climate and vegetation, we modeled remote‐sensed vegetation greenness and phenology data (Friedl et al., 2010; Gray et al., 2019) as functions of the climate covariates for a subset of years (2001–2018) for which both data sets were available.

## 2 METHODS

### 2.1 Bird data

**FIGURE 2:**
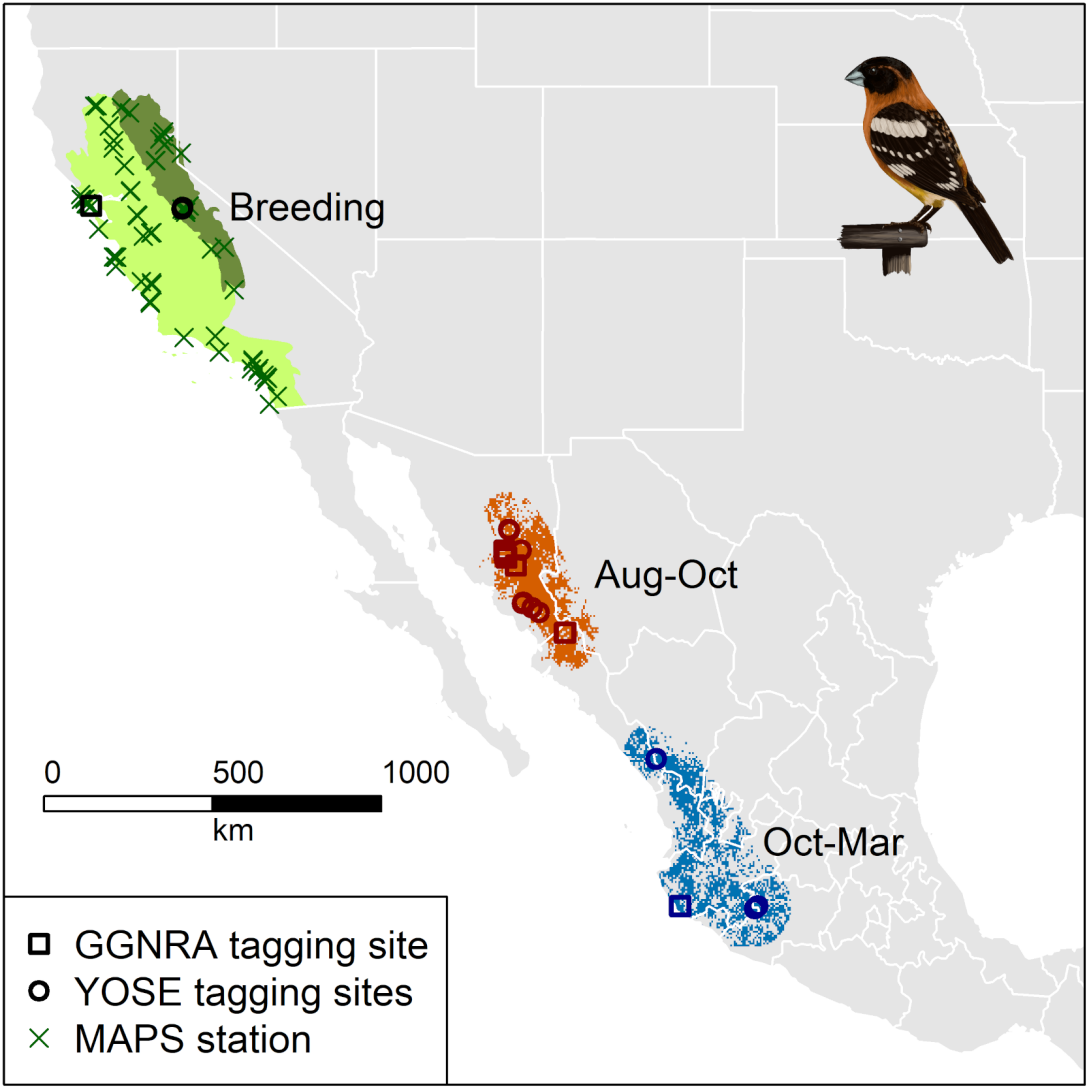
Distribution of black‐headed grosbeaks that breed in two U. S. Bird Conservation Regions (Coastal California [light green] and the Sierra Nevada [darker green]) across the annual cycle. GPS‐tagged birds were recaptured at sites in Yosemite National Park (YOSE; three birds, two MAPS stations) and Golden Gate National Recreation Area (GGNRA; one bird). Locations of these birds during the non‐breeding season were separated into two distinct periods. Molting locations of individuals (more than one if they moved within‐season) are indicated by dark orange shapes (circles for Yosemite, squares for Marin) within an orange molting range defined as a shrub and forest habitat‐filtered minimum convex polygon +0.5‐degree buffer surrounding August through mid‐October locations. Overwintering locations are indicated by blue shapes (per above, based on region, with only three birds with winter data) within a similarly defined blue region surrounding late October through March locations (Artwork by L. Helton.)

For the tracking study, we captured 33 adult grosbeaks as part of regular MAPS station operations or by luring birds into mist nets using vocalization playback at two stations in Yosemite National Park (18 birds) and at or near three stations on National Park Service properties in Marin County, California (Point Reyes National Seashore and Golden Gate National Recreation Area; 15 birds), between 2014 and 2017. Each bird was fitted with an archival GPS tag (Pinpoint 8, Lotek Wireless, Inc., Newmarket, ON, Canada) secured with Teflon or Stretch Magic jewelry cord leg harness (Rappole & Tipton, 1991), a United States Geological Survey (Biological Resources Division) numbered aluminum leg band, and a plastic color band. GPS tags, together with harnesses and leg bands, weighed slightly under 2 g (~4% of body mass). GPS tags were programmed to record a location every 4–40 days, depending on the year the tag was deployed and stage in the annual cycle, from late August through at least March. Tags were recovered from four males (three from two stations in Yosemite National Park [YOSE] and one from a station in Golden Gate National Recreation Area [GGNRA]). We defined molting and wintering ranges based on a 0.5° buffer around a minimum convex polygon enclosing molting (Aug to mid‐Oct) and wintering (mid‐Oct to Mar) GPS point locations of the four geo‐tracked birds (Figure 2). We restricted range delineation to natural shrubland and forested habitat types used by tracked black‐headed grosbeaks, as determined from GPS point locations overlaid on a 300‐m‐resolution European Space Agency Climate Change Initiative Land Cover map for 2015 (habitat classes 40, 60, and 90; Defourny et al., 2017). All field data were collected under US federal bird banding permits and relevant park or state permits and in compliance with the Guidelines to the Use of Wild Birds in Research (http://www.nmnh.si.edu/BIRDNET/guide/).

For the integrated population model, we used bird monitoring data from 44 bird‐banding stations in the Sierra Nevada (9 stations) and Coastal California (35 stations) Bird Conservation Regions (BCRs 15 and 32; https://nabci‐us.org/resources/bird‐conservation‐regions‐map/) that were operated by The Institute for Bird Populations, Point Blue Conservation Science, and 24 other cooperators following MAPS protocols (DeSante et al., 2004; Table S1). We only included data from stations where black‐headed grosbeak was documented as a breeding species, that were operated in ≥4 years, and that banded an average of ≥1 adult black‐headed grosbeak per year. All stations were operated during the breeding season (May 1 to Aug 9). Captured birds were aged as adults (after hatching year) or juveniles (hatching year). To the extent possible, adults were further microaged as either yearlings (second year birds) or older adults (after second year birds) based on criteria in Pyle (1997; Figure S1). Our analysis included 880 year‐unique captures of adult birds in the Sierra Nevada region and 2387 year‐unique captures of adult birds in the Coastal California region. Of adult birds, 70% were microaged as either yearlings or older adult birds.

### 2.2 Climate and vegetation data

To characterize annual climate variation and to link climate to demographic rates, we extracted point‐level data from the ClimateNA database ver. 7.00 (Wang et al., 2016) for the 44 breeding sites (i.e., MAPS stations) and for 1000 random points in each of the non‐breeding ranges. To control for spatial variation in climate covariates related to geographic gradients and habitat, we used differences between annual values and mean values for each point for the 1971–2000 normal period (referred to as deviations, hereafter). We selected climate covariates based on our preconceived ideas that water availability would be a strong predictor of annual variation in habitat quality and its effects on avian demographics. To characterize drought conditions on breeding, molting, and wintering grounds, we used Hargreave's climatic moisture deficit (CMD; Hargreaves & Samani, 1982), calculated as the difference between atmospheric evaporative demand and precipitation. Note that more positive CMD values indicate more severe drought. For the breeding grounds, we used summed CMD values from the overwintering period (Oct–Apr) prior to the grosbeaks’ arrival on breeding grounds, which is the period when most annual precipitation occurs in these two California regions; for the molting grounds, we used summed CMD values from Apr to Sep, encompassing a 6‐month time window spanning a period prior to and including typical monsoon months when black‐headed grosbeaks were present in that region; for wintering grounds, we summed CMD from April of the previous year to March of the current year. For molting grounds, we also calculated a ratio of early season (Jun and Jul) to late season (Aug–Sep) rainfall, to broadly characterize monsoon phenology.

To understand linkages between climate and vegetation, we extracted vegetation phenology and greenness data from the Land Cover Dynamics MCD12Q2 v.6 data product (Friedl & Sulla‐Menashe, 2019; Gray et al., 2019), which is based on 0.5‐km‐resolution Moderate Resolution Imaging Spectroradiometer (MODIS) data from the NASA Terra satellite (http://terra.nasa.gov/). The MCD12Q2 v.6 data product uses the two‐band Enhanced Vegetation Index (EVI) as the basis of phenology and greenness metrics; EVI is a composite metric that incorporates structural and seasonal components of vegetation greenness (Glenn et al., 2008; Potithepa et al., 2010). We considered two aspects of vegetation dynamics to encapsulate overall greenness and phenology. To quantify greenness, we used the integrated 8‐day interval EVI over the main growing season (EVI area). To measure phenology, we used EVI maturity date, defined as the estimated date at which 90% of the EVI amplitude was attained. We derived site‐specific annual deviations from mean values for all covariate values to account for spatial variation in overall timing and greenness. We fit linear models between mean EVI area and maturity responses and each mean climate covariate value, assuming normal responses, to assess climate effects on vegetation greenness and phenology.

### 2.3 Integrated population models

We developed a Bayesian integrated population model (IPM) based on models of age‐specific capture data and capture–mark–recapture data from MAPS stations and climate covariates. As a first step, we estimated annual indices of adult abundance for the two breeding regions based on a model of adult captures, denoted as ${\text{AHY}}_{j,k,t}$. We assumed adult captures at station *j*, region *k*, and year *t* were distributed as ${\text{AHY}}_{j,k,t}∼\text{NegBin}\left(r,p_{j,k,t}\right)$, where *r* and $p_{j,k,t}$ relate to the mean count, $\mu_{\text{AHY}\left[j,k,t\right]}$, based on $p_{j,k,t}=r/r+\mu_{\text{AHY}\left[j,k,t\right]}$. We then modeled the mean parameter, $\mu_{\text{AHY}\left[j,k,t\right]}$, with the generalized linear mixed model:

$$\log\left(\mu_{\text{AHY}\left[j,k,t\right]}\right)=\beta_{0\left[k\right]}+\beta_{1}{\text{ef}}_{j,t}+{\text{yr}}_{k,t}+{\text{sta}}_{j},$$

where $\beta_{0\left[k\right]}$ are fixed region‐specific intercepts; $\beta_{1}$ is the regression coefficient for an effort ${\text{ef}}_{j,t}$ effect (summed net‐hours relative to average net‐hours for the station); ${\text{yr}}_{k,t}$ is a random region × year effect distributed as $\text{Norm}\left(0,\sigma_{k}^{2}\right)$; and ${\text{sta}}_{j}$ is a random station effect distributed as $\text{Norm}\left(0,\sigma^{2}\right)$. We then derived the adult abundance index as $\hat{{\text{ad}}_{k,t}}=e^{\beta_{0\left[k\right]}+{\text{yr}}_{k,t}}$, which was used to inform the population state of the IPM based on means and standard deviations of posterior MCMC samples:

$$\bar{\log\left(\hat{{\text{ad}}_{k,t}}\right)}∼\text{Norm}\left(\log\left(n_{k,t}\right),s_{\log\left(\hat{{\text{ad}}_{k,t}}\right)}^{2}\right),$$

where the $n_{k,t}$ represents the population state variable of the IPM, derived as the sum of the numbers of survivors, ${\text{nsurv}}_{k,t}$, and the numbers of new recruits, ${\text{nrecr}}_{k,t}$. For the initial year (*t* = 1992), we assigned vague prior distributions for ${\text{nsurv}}_{k,1992}$ and ${\text{nrecr}}_{k,1992}$. For additional time steps (*t* > 1992), we modeled numbers of survivors and recruits as first‐order Markovian gamma random variables dependent on the previous year's population state (shape parameter), and survival (*ϕ*) and recruitment rate (*γ*) parameters (scale parameters):

$${\text{nsurv}}_{k,t}∼\Gamma \left(n_{k,t‐1},\phi_{k,t‐1}\right)$$

and

$${\text{nrecr}}_{k,t}∼\Gamma \left(n_{k,t‐1},\gamma_{k,t‐1}\right).$$

Numbers of survivors, recruits, and the recruitment rate parameter were partially informed by a model estimating the proportion of yearling adults in the population based on methods of Pyle et al. (2020). We assumed the probability of adult bird *i* at station *j* and region *k* in year *t* being a yearling bird to be distributed as a Bernoulli random variable, $Y_{i,j,k,t}∼\text{Bern}\left(pY_{i,j,k,t}\right)$, with the probability parameter modeled with the logit‐linear model: $\text{logit}\left(pY_{i,j,k,t}\right)=\beta_{0\left[k\right]}+{\text{yr}}_{k,t}+{\text{sta}}_{j}$. We derived estimates of the proportions of yearling recruits for each region and year based on back‐transforming the region × year components of the model: $\hat{pY_{k,t}}=e^{\beta_{0\left[k\right]}+{\text{yr}}_{k,t}}/\left(1+e^{\beta_{0\left[k\right]}+{\text{yr}}_{k,t}}\right)$ and estimates of the numbers of new yearling recruits as $\hat{\text{ad}1_{k,t}}=\hat{pY_{k,t}}\times \hat{{\text{ad}}_{k,t}}$ and numbers of surviving adults as $\hat{\text{ad}2_{k,t}^{+}}=\hat{\text{a}{\text{d}}_{k,t}}‐\hat{\text{ad}1_{k,t}}$. These estimates informed the recruitment and survival components of the population dynamics model:

$$\bar{\hat{ad1_{k,t}}}∼Norm{\left.\right|}_{0}^{\infty }\left(nrecr_{k,t},s_{\hat{ad1_{k,t}}}^{2}\right)$$

and

$$\bar{\hat{ad{2^{+}}_{k,t}}}∼Norm{\left.\right|}_{0}^{\infty }\left(nsurv_{k,t},s_{\hat{a{d2^{+}}_{k,t}}}^{2}\right).$$

Additionally, we derived an observed recruitment variable, $\hat{g_{k,t}}=\hat{{ad1}_{k,t+1}}/\hat{{ad}_{k,t}}$, which we also assumed its posterior mean to be distributed as $Norm{|}_{0}^{\infty }\left(\gamma_{k,t},S_{\hat{g_{k,t}}}^{2}\right)$ to inform the recruitment rate parameter.

The survival probability parameter of the population dynamics model, $\phi_{k,t}$, was informed by a state–space version of the Cormack–Jolly–Seber (CJS) model that accounts for transients (i.e., individuals that permanently emigrate after initial capture; Saracco et al., 2012) applied to CMR data from *i* = 1, …, *M* adult birds captured at MAPS stations. We modeled covariate effects based on generalized linear mixed models:

$$\text{logit}\left(\phi_{i,j,k,t}\right)=\beta_{0\left[k\right]}+\beta_{1‐4\left[k\right]}X+{\text{yr}}_{k,t}$$

and

$$\log\left(\gamma_{k,t}\right)=\beta_{0\left[k\right]}+\beta_{1‐4\left[k\right]}X+{\text{yr}}_{k,t}.$$

Model terms included intercepts ($\beta_{0[k]}$), regional covariate effects ($\beta_{1‐4[k]}$), a matrix, **X**, that included four climate covariates, each standardized for analysis to mean = 0, SD = 1, and zero‐mean random region × year effects (${\text{yr}}_{k,t}$). Climate covariates included: (1) drought conditions on breeding grounds (station‐scale CMD deviation for Oct–Apr; CMD_b), (2) average drought conditions on wintering grounds (mean Apr–Mar CMD deviation across winter ground samples; CMD_w), (3) average drought conditions on molting grounds (Apr–Sep) after accounting for correlation with winter drought conditions (residuals of a regression of molting grounds drought on wintering grounds drought), and (4) the ratio of early (Jun–Jul) to late (Aug–Sep) rainfall deviation on molting grounds (ELR_m). We used CMD_m_res, rather than the original CMD deviation variable for the molting grounds, as it was moderately correlated with CMD_w (*r* = .62) and an initial analysis of the survival model suggested a stronger relationship for winter drought than for molting grounds drought. Thus, CMD_m_res can be interpreted as drought effects on molting grounds independent of drought effects common to both winter and molting grounds (Dormann et al., 2013; Graham, 2003).

Additional parameters in the CJS models included residency probability, $\pi_{i,j,k,t}$, and parameters describing the observation process, capture probability, $p_{i,j,k,t}$, and probability of predetermining a newly banded bird as a resident bird, $\rho_{i,j,k,t}$ (i.e., recapturing a bird ≥7 days apart in the season it was banded; Saracco et al., 2012). For residency probability, we defined a logit‐linear model with random region × year effects; for the observation parameters, we defined logit‐linear models that included intercepts and zero‐mean random station effects.

We implemented all models using JAGS (Plummer, 2003) in R (R Core Team, 2021) using the jagsUI package (Kellner, 2021). We assigned $\text{Norm}\left(0,100\right)$ priors to regression coefficients and fixed region intercepts and $\text{U}\left(0,1\right)$ priors to inverse‐logit transformed intercepts in the CJS model. Posterior inferences were based on three independent Markov chain Monte Carlo (MCMC) simulations of 40,000 iterations after adaptive and burn‐in phases of 20000 each and thinning by 5 to reduce chain autocorrelation. Gelman–Rubin statistic values <1.01 for all model parameters suggested successful model convergence (Gilks et al., 1996), and Bayesian *p*‐values from posterior predictive checks (*χ* ^2^ test for age‐specific capture models; Tukey–Freeman test for CJS model) suggested adequate model fits (.3 < *p* < .5; Conn et al., 2018). We summarized posterior parameter estimates as 89% credible intervals around median values, and for regression coefficients, we provide the probability that parameter estimates are < (for negative effect estimates) or > (for positive effect estimates) zero (McElreath, 2020).

We derived estimates of regional annual population change from posterior distributions as: $\hat{\lambda_{k,t}}=n_{k,t+1}/n_{k,t}$. To estimate population trends that incorporate uncertainty associated with annual variation, we calculated the geometric mean of the $\hat{\lambda_{k,t}}$ for each MCMC iteration. In addition, we assessed the association between recruitment and survival and between numbers of survivors and numbers of recruits by calculating Pearson's correlation coefficients for each MCMC iteration. We assessed relative contribution of adult survival to population growth based on the proportion of population change explained by the adult apparent survival probability (i.e., $\hat{\phi }/\hat{\lambda }$; Nichols et al., 2000). Data and R‐code to reproduce results are available in an open archive hosted by the Open Science Framework (https://doi.org/10.17605/OSF.IO/YAU92).

## 3 RESULTS

### 3.1 Movement and connectivity

All four GPS‐tagged birds for which we recovered data were males and showed similar between‐season movements (Figure 2) despite representing geographically distinct breeding sites, different years, and including both yearling and older birds (Table 1). Movements between breeding sites and (presumed) molting locations in northwestern Mexico occurred in early to mid‐August and averaged ~1300 km. Movements between molting and overwintering sites occurred in mid‐to‐late October and were more variable with the two older YOSE birds showing the shortest (730 km) and longest (1325 km) observed molt winter movements, and the GGNRA bird moving an intermediate distance (869 km; data only available for three of the four birds). Within‐season movements were common, particularly during the molting season for the yearling birds, which ranged from a few kilometers to hundreds of kilometers near seasonal transitions. Vegetation greenness early in the molting season was similarly low at sites occupied for the yearling birds (EVI ~ 0.4) compared to sites occupied by the older males (EVI ~ 0.6; Figure S1). While the range of dates spanned and resolution of the archived location data was greater for the two yearling birds (5–10 days) compared to the older birds (16–40 days), both younger birds made large‐scale movements across a time period during which the older birds appeared to be largely stationary (Sep 5 to Oct 15).

**TABLE 1:** Movements of four male GPS‐tagged black‐headed grosbeaks from breeding grounds in Yosemite National Park (YOSE) or Golden Gate National Recreation Area (GGNRA), California

Bird ID	Age at tagging (years)	Year	Movement distance (km)			
			Breed → Molt	Within molt (Aug 8 to Oct 15)	Molt → Winter	Within winter (Oct 16 to Mar 24)
YOSE‐1	9	2014–15	1300	3	1325	12
YOSE‐2	12+	2014–15	1369	−	730	3
YOSE‐3	1	2017	1117	393, 3, 31, 26, 26	NA	NA
GGNRA‐1	1	2017–18	1527	4, 10, 10, 37, 242, 2	869	1

Note

Details of movements of YOSE‐1 were reported in Siegel et al. (2016). Movement data following the molting season were not available (NA) for YOSE‐3. Only movements >1 km are shown.

### 3.2 Climate variation and relationship to vegetation dynamics

**FIGURE 3:**
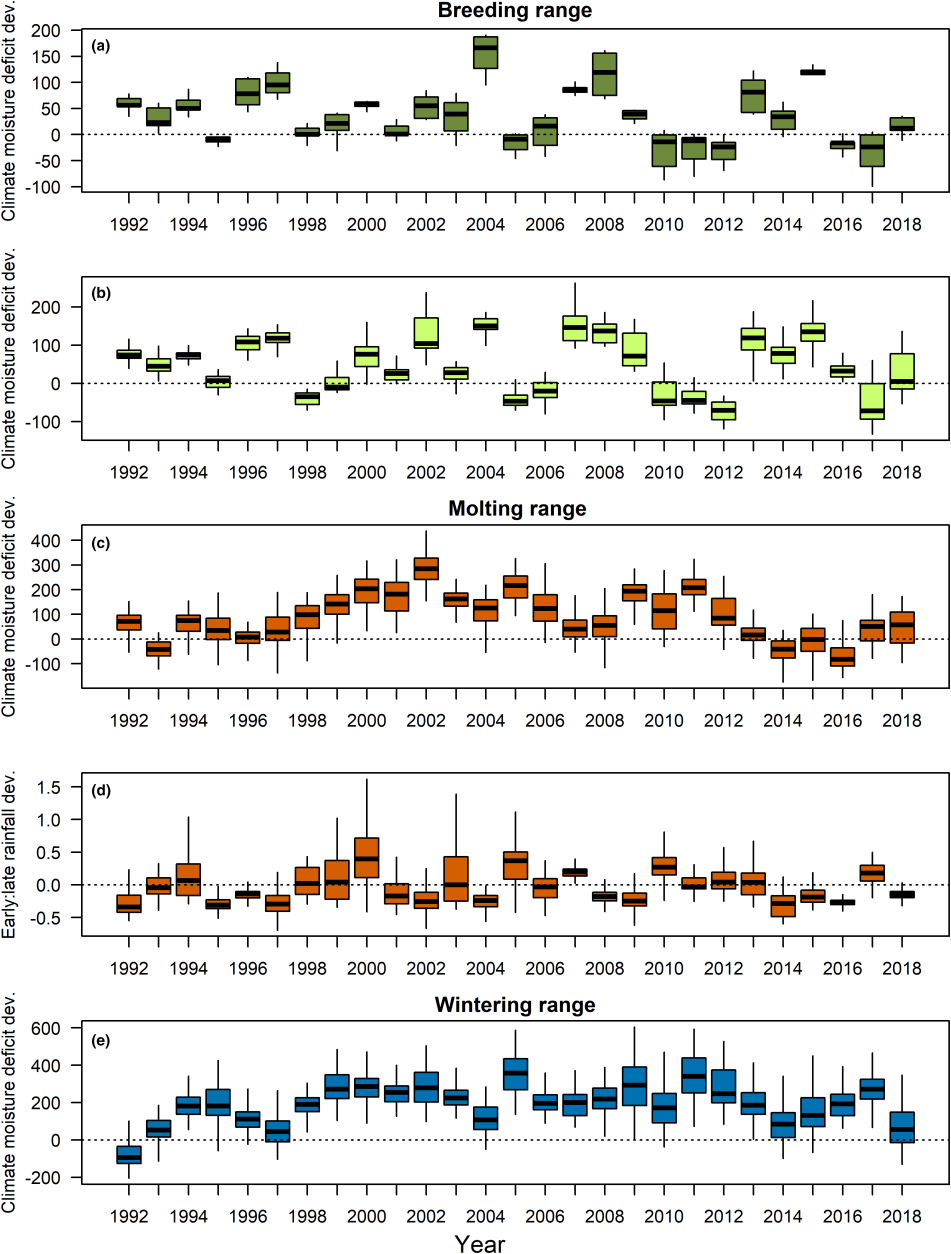
Temporal variation in climate variables, 1992–2018, from breeding sites (Monitoring Avian Productivity and Survivorship [MAPS] stations in (a) the Sierra Nevada and (b) Coastal California) and from random sites on molting (c, d) and wintering (e) ranges

Oct–Apr climatic moisture deficit deviation on the breeding grounds was highly variable among years with drought conditions tending to dominate the time series (median CMD >0 in 19 of 27 years [70%]; Figure 3a,b). For molting and wintering regions, drought conditions were even more common, with median Apr–Sep CMD deviation >0 in 24 of 27 years (89%) on molting grounds (Figure 3c) and Apr–Mar CMD deviation >0 in 26 of 27 years (96%) on wintering grounds (Figure 3e). The ratio of early (Jun–Jul) to late (Aug–Sep) season rainfall on molting grounds did not show any clear temporal pattern (Figure 3d).

**FIGURE 4:**
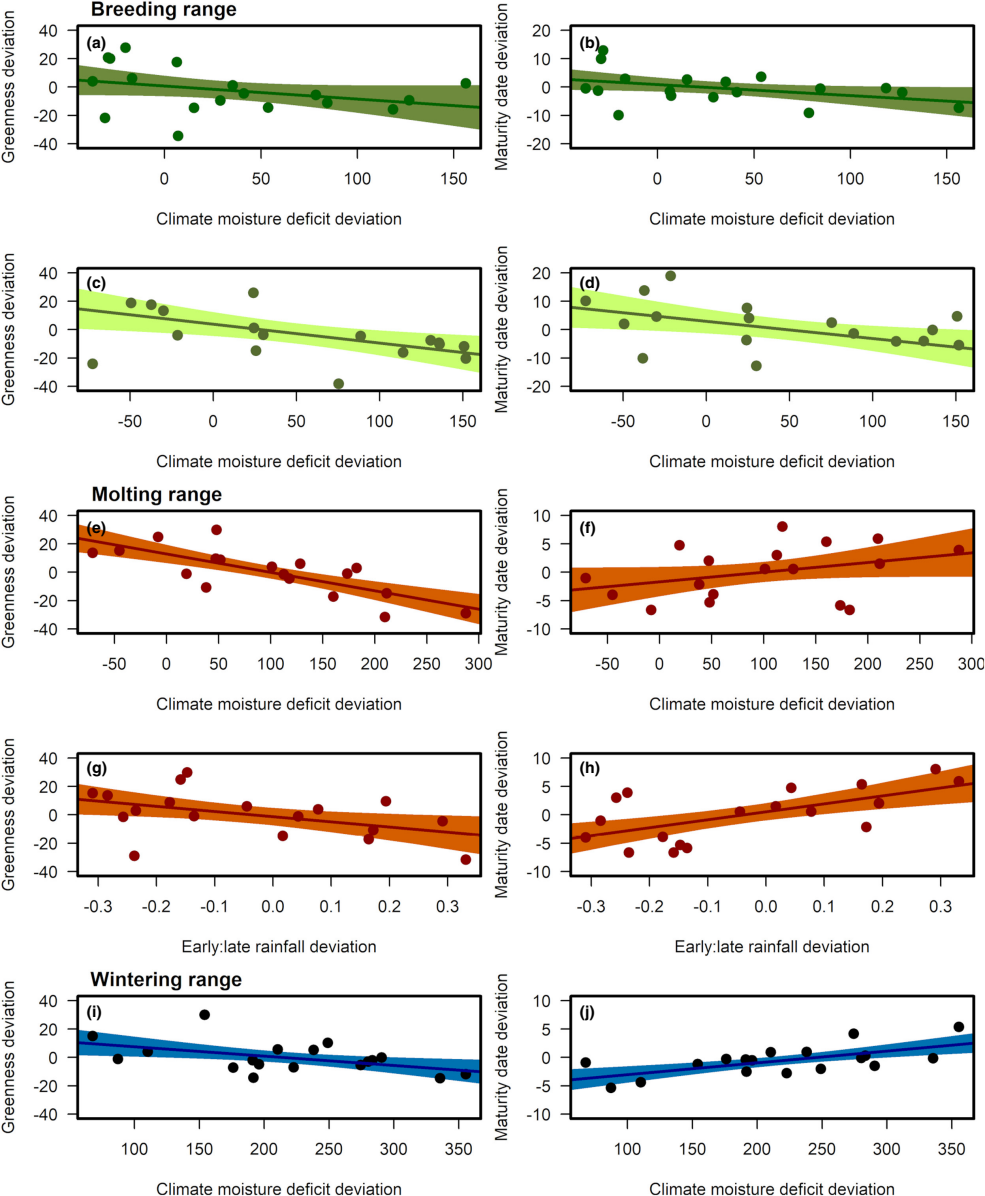
Relationships between climate covariates and vegetation greenness and maturity dates (2001‐2018) across the annual cycle. Climate covariates included climate moisture deficit deviation on breeding grounds (a, d), climate moisture deficit deviation on molting grounds (e, f), rainfall timing on molting grounds (early:late rainfall deviation; g, h), and climate moisture deficit deviation on wintering grounds (i, j). Fit lines are from linear regressions and shaded regions show 89% credible intervals. Breeding season variables were averaged across MAPS stations separately for the Sierra Nevada (a, b) and Coastal California (c, d)

Vegetation greenness area deviation averaged across breeding sites tended to be negatively related to Oct–Apr drought for both the Sierra Nevada ($\hat{\beta }$ = −5.42, 89% CI: [−12.10, 1.26], $p\left(<0\right)$ = .91; Figure 4a) and Coastal California sites ($\hat{\beta }$ = −10.03, 89% CI: [−17.59, −2.48], $p\left(<0\right)$ = .98; Figure 4c). That is, in both breeding regions, drier years were associated with less vegetation greenness. Vegetation also matured earlier in years of more severe drought at Sierra Nevada ($\hat{\beta }$ = −2.28, 89% CI: [−4.60, 0.03], $p\left(<0\right)$ = .94; Figure 4b) and Coastal California ($\hat{\beta }$ = −4.62, 89% CI: [−8.52, −0.81], $p\left(<0\right)$ = .97; Figure 4d) breeding sites.

Vegetation on molting grounds was greener in cooler wetter years ($\hat{\beta }$ = −12.29, 89% CI: [−17.11, −7.53], $p\left(<0\right)$ = 1.00; Figure 4e), and, in contrast to the pattern observed on the breeding grounds, vegetation matured later in years with more severe drought ($\hat{\beta }$ = 1.64, 89% CI: [−0.28, 3.55], $p\left(>0\right)$ = .92; Figure 4f). When proportionally more rain fell earlier in the season, vegetation tended to be less green ($\hat{\beta }$ = −6.18, 89% CI: [−12.79, 0.56], $p\left(<0\right)$ = .93; Figure 4g) and matured later ($\hat{\beta }$ = 2.86, 89% CI: [1.24, 4.46], $p\left(>0\right)$ = 1.00; Figure 4h).

Vegetation greenness area deviation on wintering grounds followed the same pattern as that seen on the molting grounds: drier years were less green ($\hat{\beta }$ = −5.27, 89% CI: [−9.45, −1.11], $p\left(<0\right)$ = .98; Figure 4i) and with earlier maturing vegetation ($\hat{\beta }$ = 1.66, 89% CI: [0.79, 2.54], $p\left(>0\right)$ = 1.00; Figure 4j).

### 3.3 Population dynamics and demography

**FIGURE 5:**
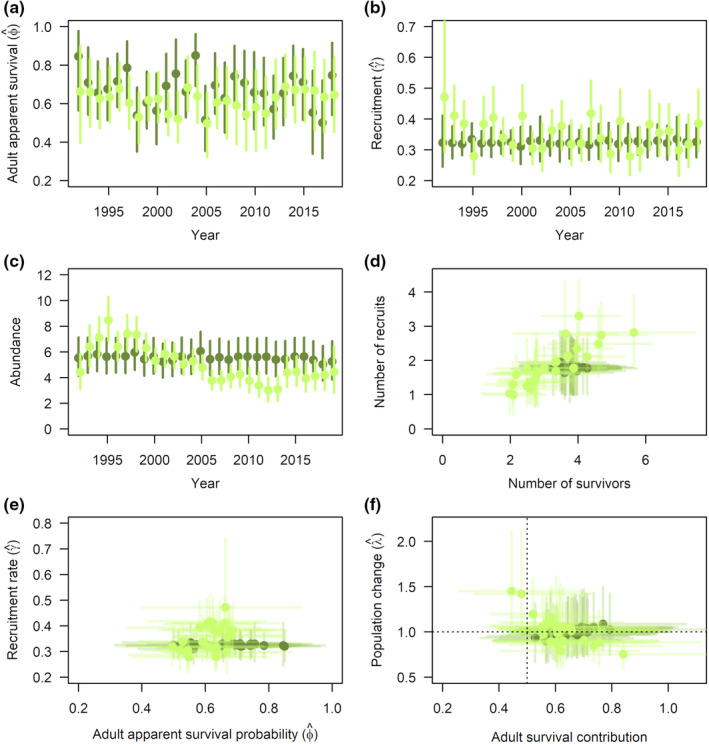
Annual adult apparent survival probabilities, $\hat{\phi }$ (a), recruitment rates, $\hat{\gamma }$ (b), abundance indices (c), demographic correlations (d, e), and demographic contributions to population change, $\hat{\phi }/\hat{\lambda }$ (f). Median values ±89% credible intervals are shown. Darker green points = Sierra Nevada; light green points = Coastal California

Adult apparent survival probabilities varied substantially among years, particularly for the Sierra Nevada populations (Figure 5a). Recruitment rates were also annually variable for Coastal California but relatively stable across years for the Sierra Nevada (Figure 5b). Average demographic rates were similar between the two regions with adult survival slightly higher for the Sierra Nevada ($\hat{\phi_{0}}$ = 0.69, 89% CI: [0.63, 0.77]) compared to Coastal California ($\hat{\phi_{0}}$ = 0.62, 89% CI: [0.57, 0.69]) and recruitment rate slightly lower for the Sierra Nevada ($\hat{\gamma_{0}}$ = 0.32, 89% CI: [0.30, 0.35]) compared to Coastal California ($\hat{\gamma_{0}}$ = 0.35, 89% CI: [0.32, 0.38]).

Coastal California populations increased early in the study, peaking in 1995, and then declining until 2005, after which time population size remained relatively consistent and similar to abundance levels seen at the beginning of the time series (Figure 5c). Sierra Nevada populations were more consistent across the study period. Neither region showed long‐term trend; annual population change averaged across models was −0.19% × year^−1^ (89% CI: [−1.67, 1.32]) for the Sierra Nevada region and −0.01% × year^−1^ (89% CI: [−1.94, 1.96]) for Coastal California.

The estimated number of recruits was similar across years for the Sierra Nevada region, but highly variable among years for Coastal California. Numbers of survivors and recruits tended to be negatively correlated for the Sierra Nevada region (posterior sample correlations *r* = −.23, 89% CI: [−0.51, 0.09], $p\left(>0\right)$ = .88) and were positively correlated for the Coastal California region (*r* = .42, 89% CI: [0.15, 0.64], $p\left(>0\right)$ = .99; Figure 5d). Adult apparent survival probability showed little evidence of correlation with recruitment rate for the Sierra Nevada (*r* = −.01, 89% CI: [−0.58, 0.59], $p\left(<0\right)$ = .51), and weak positive correlation for Coastal California (*r* = .16, 89% CI: [−0.40, 0.63], $p\left(>0\right)$ = .68; Figure 5e). Contributions of adult survival to annual population change ($\hat{\phi }/\hat{\lambda }$) were mostly greater than contributions of recruitment to population change ($\hat{\phi }/\hat{\lambda }$ = 0.66, 89% CI: [0.43, 0.97]) for the Sierra Nevada ($\hat{\phi }/\hat{\lambda }$ = 0.61, 89% CI: [0.39, 0.92]) and for Coastal California (Figure 5f); however, recruitment contributed more to population change in years of increase, particularly in years of high population growth early in the time series in Coastal California (1992–1993 and 1994–1995).

### 3.4 Climate–demographic rate relationships

**FIGURE 6:**
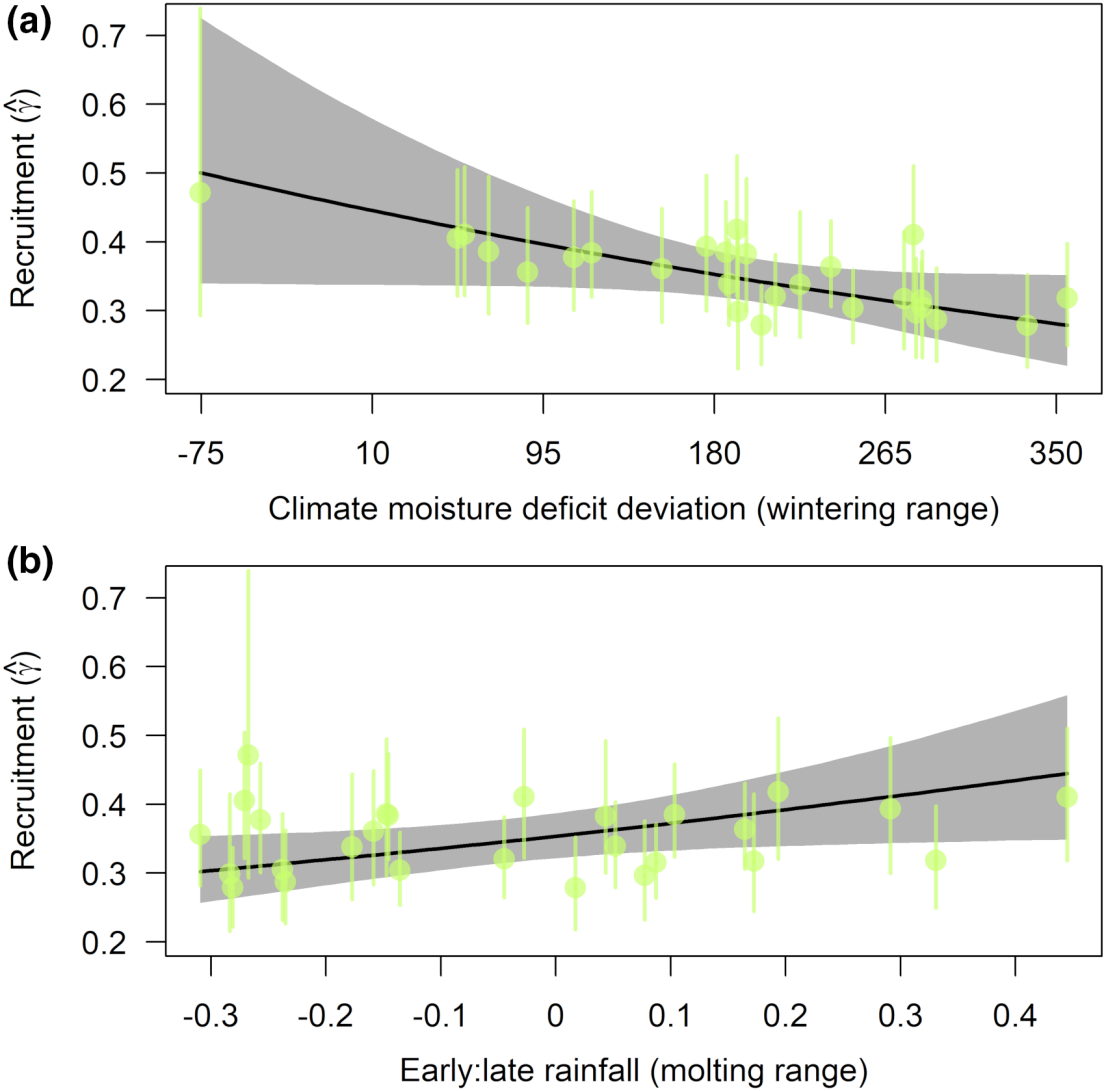
Relationships between recruitment rate in the Coastal California region and drought conditions on the wintering range (a) and ratio of early‐to‐late season rainfall on the molting range (b)

We found only weak support for most demographic rate–climate relationships that we considered (Table 2). Effect sizes, in several cases, were of a magnitude that could have large consequences on population size; however, precision of estimates was low, precluding strong inferences on climate effects on survival. We found strongest evidence of covariate effects with respect to recruitment rates for the Coastal California populations. Recruitment in this breeding region was higher following relatively cool and wet years on the wintering grounds (lower CMD_w; Figure 6a) and years with relatively more rainfall early in the Monsoon season on the molting grounds (ELR_m; Figure 6b).

**TABLE 2:** Effect estimates (median $\hat{\beta }$ [89% CI]) and probabilities that estimates are >0 (for positive estimates) or <0 (for negative estimates) for climate covariates included in logit‐linear model of adult survival ϕ and log‐linear model of recruitment γ

Covariate	Sierra Nevada		Coastal California	
	ϕ effect	γ effect	ϕ effect	γ effect
Drought, breeding grounds (CMD_b)	0.23 (−0.26, 0.80) *p*(<0) = .77	−0.01 (−0.12, 0.09) *p*(<0) = .58	−0.01 (−0.31, 0.27) *p*(<0) = .53	0.06 (−0.05, 0.17) *p*(<0) = .81
Drought, wintering grounds (CMD_w)	−0.14 (−0.66, 0.28) *p*(<0) = .69	0.01 (−0.08, 0.10) *p*(>0) = .58	−0.14 (−0.55, 0.22) *p*(<0) = .72	−0.13 (−0.27, 0.00) *p*(<0) = .93
Drought residuals, molting grounds (CMD_m_res)	0.18 (−0.17, 0.57) *p*(>0) = .79	0.00 (−0.07, 0.07) *p*(>0) = .51	−0.11 (−0.41, 0.17) *p*(<0) = .74	0.01 (−0.10, 0.12) *p*(<0) = .56
Early:late rainfall, molting grounds (ELR_m)	−0.19 (−0.61, 0.23) *p*(<0) = .77	−0.02 (−0.10, 0.06) *p*(<0) = .63	0.03 (−0.31, 0.33) *p*(>0) = .57	0.11 (0.01, 0.21) *p*(>0) = .96

## 4 DISCUSSION

Our study provides a model of how tracking, environmental, and demographic data can inform temporal variation in habitat quality and patterns of population change at regional scales. Although based on movements of just four individuals, our tracking data showed consistent between‐season movements, supporting the pattern reported by Siegel et al. (2016) of distinct molting and wintering locations for California breeding populations of black‐headed grosbeak. While we found that birds from our two breeding regions shared molting and wintering regions, climate variation across the annual cycle appeared to have unique effects on the demography and dynamics of populations in each breeding region. There was no long‐term population trend for either breeding region; however, the decline in Coastal California after the mid‐1990s matched that reported from point count data from this region (−2%/year; Dettling et al., 2021). The Sierra Nevada populations appeared to be more tightly regulated with relatively little annual variation and numbers of recruits and survivors tending to be negatively correlated.

Our tracking data suggested that within‐season movements outside of the breeding season may be common for black‐headed grosbeaks that breed in California, particularly during the molting period, although larger movements near seasonal transitions may more accurately be considered to be protracted migrations than within‐season movements. Support for non‐breeding season movements has been reported for other migratory landbird species (Cormier et al., 2013; Mancuso et al., 2021; Ruiz‐Gutierrez et al., 2016; Stutchbury et al., 2016), and flexibility to move to new sites within seasons may be a critical adaptation for tracking uncertain environmental conditions. Both yearling males that showed within‐molting season movements initially settled in habitats that were less green than those settled in by the older adults. It is possible that older males exclude younger males from preferred greener habitat. However, apparent age differences could reflect alternative habitat selection strategies with younger birds selecting multiple habitats that green up at different rates (Figure S2). Patterns of habitat use may also vary among years with habitat breadth expanding or contracting depending on conditions (Pyle et al., 2009). Finally, apparent age differences could also simply reflect lower availability of greener habitats in the year the yearlings were tracked (2017) compared to the year the older adults were tracked (2015). Clearly, additional data across age and sex classes across multiple years will be needed to fully elucidate non‐breeding habitat needs.

We found clear signals of climate variation on habitat, with greenness and maturity date dependent on drought conditions at sites across the annual cycle and on rainfall timing at sites on molting grounds. In all seasons, more severe drought was associated with less greenness. Trends in drought across the black‐headed grosbeak breeding range suggest that more severe and multi‐year drought years are becoming more common, which will likely have implications for habitat quality (Breshears et al., 2005; Diffenbaugh et al., 2015; Goulden & Bales, 2019; Trujillo et al., 2012; Williams et al., 2020). Monsoon precipitation on molting grounds may also be declining (Pascale et al., 2017) and becoming more spatially (Demaria et al., 2019) and temporally (Meyer & Jin, 2017) variable, which could then affect spatiotemporal patterns in habitat variation (Méndez‐Barroso et al., 2009).

Drought conditions were also associated with vegetation phenology. More severe drought on the breeding grounds was associated with earlier maturing vegetation, while more severe drought on molting and wintering grounds was associated with later maturing vegetation. A trend toward earlier maturing vegetation on breeding grounds could yield mismatches in the migration and breeding phenology of birds (Mayor et al., 2017); however, populations may also adapt to climate trends in space and/or time, and in some circumstances demographic rates can improve under warmer, drier conditions (Saracco et al., 2019; Socolar et al., 2017). We also found evidence that rainfall timing on molting grounds influences greenness and vegetation phenology. Although there was no trend evident in the our rainfall timing metric over the years we considered here, other studies have suggested a trend toward later monsoon rainfall (Cook & Seager, 2013; Grantz et al., 2007) that may result in later maturing and less green habitats, on average, as anthropogenic climate change continues.

While we found clear vegetation responses to climate variation, we detected only weak support for grosbeak demographic responses to most climate covariates. Drought in habitats and at times of year where water is a limiting resource can adversely affect vital rates of migratory birds (Dugger et al., 2004; Rockwell et al., 2017; Saracco et al., 2018; Sillett et al., 2000). Thus, we expected drought effects on demographics might be greatest on the wintering grounds as the dry season peaks late in winter prior to spring migration and survival of young and adults may consequently suffer. While point estimates of effects of wintering grounds drought on adult survival were in the expected direction (i.e., negative effects of drought) for both breeding regions, credible intervals were broad, limiting inferences. Drought on the wintering grounds was more strongly correlated with declines in recruitment, although this relationship was limited to the Coastal California populations, which showed greater variation in recruitment and years of large population growth corresponding to high recruitment rates. Recruitment in the Coastal California region was also associated with rainfall timing, with years having relatively more rainfall early in the Monsoon season tending to have higher recruitment than in years tending to have later rainfall. Given that earlier rainfall was associated with later vegetation maturation on molting grounds, it could be that such conditions were differentially favorable for first‐year survival in such years, as young birds tend to migrate and molt later than adults (Ortega & Hill, 2020). Weak and/or sometimes conflicting relationships between demographic parameters and breeding and molting grounds climate covariates may reflect water not be a limiting resource at these life cycle phases. For example, productivity may decline for some bird species in coastal California in drought years (Chase et al., 2005); however, black‐headed grosbeaks finish breeding relatively early in that region and so may not be negatively impacted by drought, which may have stronger effects on habitats later in the season. Indeed, nest survival was higher for black‐headed grosbeaks in relatively warm years at a site on the edge of the eastern Sierra Nevada/western Great Basin (Becker & Weisberg, 2015), and overall productivity of many bird species in the western Sierra Nevada can be relatively high in warm, dry years (Saracco et al., 2019).

Recent technological advances have enabled the rapid expansion of studies identifying geographic structure and tracking movements of populations of small migratory birds across the annual cycle (Mckinnon & Love, 2018; Ruegg et al., 2014; Rushing, Ryder, Scarpignato, et al., 2016); however, a major challenge remains to better understand causes and consequences of movements on population demography, dynamics, and conservation (Ruegg et al., 2020; Rushing, Ryder, & Marra, 2016; Saracco & Rubenstein, 2020). Meeting this challenge will be critical for understanding full‐life cycle drivers of population change (Hostetler et al., 2015). We suggest that linking individual movement data to demographic monitoring data can be a valuable tool for enhancing our ability to understand drivers of broad‐scale dynamics and trends of migratory species. Existing networks of monitoring sites, such as those coordinated by the MAPS program, provide an under‐utilized but potentially efficient framework for deploying and recovering tags or obtaining biological samples and providing demographic data for such analyses. The value of these direct measures of movement and demography can be further enhanced by taking advantage of other large‐scale observational data sets, such as eBird (Sullivan et al., 2009), which can help to refine understanding of the timing and extent of seasonal movements (Fournier et al., 2017; Vincent et al., 2022).

In addition to the general approach of linking movement and demographic data, our example highlights the potential for leveraging detailed age‐specific capture data in a novel integrated population model framework to inform a regional index of average population size, as well as recruitment rates and relative contributions of new yearling recruits vs. older adults and their respective demographic rates in driving population dynamics. Our model builds on previous integrated population models for MAPS data, in which recruitment components of the model were largely latent parameters and for which spatially incongruent data informed the population state (Ahrestani et al., 2017; Saracco & Rubenstein, 2020). As in those previous efforts, years of large population increases appeared to be driven substantially by large recruitment events. Finally, although we focused here on just the survival and recruitment parameters, data on ratios of young to adult birds could also be leveraged to help decompose productivity and juvenile survival components of the recruitment process (Saracco & Rubenstein, 2020).

## AUTHOR CONTRIBUTIONS

**James F. Saracco:** Conceptualization (equal); Data curation (equal); Formal analysis (lead); Methodology (lead); Software (lead); Validation (lead); Visualization (lead); Writing – original draft (lead); Writing – review & editing (equal). **Renée L. Cormier:** Conceptualization (equal); Data curation (supporting); Funding acquisition (supporting); Investigation (equal); Methodology (supporting); Resources (supporting); Writing – review & editing (supporting). **Diana L. Humple:** Conceptualization (equal); Data curation (supporting); Funding acquisition (equal); Investigation (equal); Methodology (supporting); Writing – review & editing (supporting). **Sarah Stock:** Funding acquisition (equal); Investigation (supporting); Resources (supporting); Writing – review & editing (supporting). **Ron Taylor:** Data curation (equal); Investigation (equal); Methodology (supporting). **Rodney B. Siegel:** Conceptualization (equal); Funding acquisition (equal); Investigation (supporting); Methodology (supporting); Project administration (lead); Resources (lead); Supervision (lead); Writing – original draft (supporting); Writing – review & editing (equal).

## CONFLICT OF INTEREST

None declared.

### OPEN RESEARCH BADGES

This article has been awarded Open Data, Open Materials Badges. All materials and data are publicly accessible via the Open Science Framework at https://doi.org/10.17605/OSF.IO/YAU92 and in Movebank (movebank.org; ID: 1909247951).

## Supporting information

Supplementary Material

## Data Availability

GPS‐tagging data have been deposited in Movebank (movebank.org; ID: 1909247951). Data and R‐code to reproduce results are available in an open archive hosted by the Open Science Framework (https://doi.org/10.17605/OSF.IO/YAU92).
