# Supplementary material for: Demographic responses to climate‐driven variation in habitat quality across the annual cycle of a migratory bird species
Source: Ecol Evol. 2022 Jun 11;12(6):e8934. doi: 10.1002/ece3.8934 (PMC9188024; doi:10.1002/ece3.8934)

**Supporting information S1. Supplementary table and figures**

Table S1. Locations of Monitoring Avian Productivity and Survivorship (MAPS) stations included in analyses and summary of numbers of black-headed grosbeak individuals and recaptures at those stations.

| Station code | Station name | Number of adults | Number  of recaptures | Number of years | Mean Individuals / year | State | Latitude | Longitude |
| --- | --- | --- | --- | --- | --- | --- | --- | --- |
| Sierra Nevada (BCR 15) | | | | | | | | |
| ZUME | Zumwalt Meadow | 59 | 8 | 11 | 5.36 | CA | 36.792 | -118.596 |
| BIME | Big Meadow | 159 | 12 | 27 | 5.89 | CA | 37.705 | -119.754 |
| TAME | Tamarack Meadow | 5 | 0 | 5 | 1.00 | CA | 37.779 | -119.744 |
| HODG | Hodgdon Meadow | 264 | 74 | 28 | 9.43 | CA | 37.794 | -119.863 |
| BGOK | Big Oak Flat | 86 | 26 | 8 | 10.75 | CA | 39.058 | -120.650 |
| BIGO | Big Oak Flat 2 | 52 | 7 | 7 | 7.43 | CA | 39.058 | -120.658 |
| FROF | Little Valley | 13 | 1 | 12 | 1.08 | NV | 39.256 | -119.878 |
| CAVA | Carman Valley | 37 | 2 | 13 | 2.85 | CA | 39.700 | -120.450 |
| RARA | Ramelli Ranch | 64 | 3 | 8 | 8.00 | CA | 39.814 | -120.416 |
| Coastal California (BCR 32) | | | | | | | | |
| SM-- | Santa Margarita | 28 | 0 | 12 | 2.33 | CA | 33.267 | -117.371 |
| FALL | Naval Weapons Station Detachment Fallbrook | 31 | 4 | 6 | 5.17 | CA | 33.354 | -117.305 |
| DL-- | DeLuz | 129 | 24 | 15 | 8.60 | CA | 33.381 | -117.322 |
| CS-- | Case Springs | 37 | 3 | 6 | 6.17 | CA | 33.433 | -117.403 |
| GOBE | Gobernadora Canyon | 46 | 12 | 8 | 5.75 | CA | 33.539 | -117.589 |
| UPWE | Upper Weir Canyon | 14 | 0 | 5 | 2.80 | CA | 33.839 | -117.739 |
| SOLS | Solstice Canyon | 82 | 1 | 8 | 10.25 | CA | 34.038 | -118.746 |
| ATCK | Atascadero Creek | 38 | 4 | 5 | 7.60 | CA | 34.422 | -119.803 |
| HMWR | Hopper Mountain | 30 | 2 | 6 | 5.00 | CA | 34.458 | -118.858 |
| POWL | MBSP - Powell II | 24 | 0 | 7 | 3.43 | CA | 35.325 | -120.815 |
| CHFL | Chorro Flats | 23 | 2 | 6 | 3.83 | CA | 35.361 | -120.818 |
| SANG | Salinas River NG | 13 | 0 | 6 | 2.17 | CA | 35.860 | -120.779 |
| BSOL | Big Sur | 484 | 36 | 17 | 28.47 | CA | 36.286 | -121.842 |
| REDR | Red Rocks | 9 | 0 | 6 | 1.50 | CA | 36.523 | -121.818 |
| SCBR | Schulte Bridge | 21 | 1 | 6 | 3.50 | CA | 36.525 | -121.831 |
| CARM | Carmel River Mouth | 36 | 9 | 13 | 2.77 | CA | 36.537 | -121.913 |
| RASC | Rancho San Carlos | 9 | 1 | 6 | 1.5 | CA | 36.537 | -121.871 |
| OFWA | O'Neill Forebay Wildlife Area | 25 | 5 | 10 | 2.5 | CA | 37.080 | -121.022 |
| SOSA | South San Joaquin | 46 | 6 | 10 | 4.6 | CA | 37.176 | -120.761 |
| HONC | TomKat (Honsinger Creek) | 22 | 1 | 8 | 2.75 | CA | 37.266 | -122.360 |
| CISL | Christman Island | 27 | 2 | 5 | 5.4 | CA | 37.614 | -121.184 |
| GACO | Gardner's Cove | 6 | 0 | 6 | 1 | CA | 37.637 | -121.209 |
| REDC | Redwood Creek | 47 | 10 | 18 | 2.61 | CA | 37.867 | -122.579 |
| PIGU | Pine Gulch Creek | 28 | 7 | 26 | 1.08 | CA | 37.919 | -122.690 |
| PALO | Palomarin | 64 | 7 | 27 | 2.37 | CA | 37.931 | -122.740 |
| MUHO | Muddy Hollow | 40 | 3 | 24 | 1.67 | CA | 38.048 | -122.870 |
| LACR | Lagunitas Creek | 26 | 8 | 16 | 1.63 | CA | 38.060 | -122.771 |
| TAFO | Tall Forest | 28 | 5 | 11 | 2.55 | CA | 38.255 | -121.404 |
| WELE | Wendell's Levee | 132 | 19 | 12 | 11.00 | CA | 38.267 | -121.394 |
| BOBE | Bobelaine | 31 | 0 | 6 | 5.17 | CA | 38.925 | -121.581 |
| SN-- | Sul Norte | 100 | 18 | 6 | 16.67 | CA | 39.386 | -121.922 |
| STCR | Stony Creek | 203 | 49 | 11 | 18.45 | CA | 39.590 | -121.907 |
| OHM- | Ohm | 87 | 2 | 9 | 9.67 | CA | 39.963 | -122.046 |
| SADA | Saeltzer Dam | 57 | 12 | 8 | 7.13 | CA | 40.494 | -122.479 |
| PRAR | Project Area | 72 | 7 | 10 | 7.20 | CA | 40.504 | -122.403 |

Figure S1. Wings of older (after-second-year) adult black headed grosbeaks show uniform flight feathers and wing coverts typical of a complete prebasic molt (a). Younger birds (b) show contrast between fresher replaced formative wing coverts and retained juvenile primary coverts and flight feathers. Both birds shown here are males for which aging is relatively straightforward because of the high contrast between replaced black and retained brown feathers; however, the same ageing criteria apply to females.


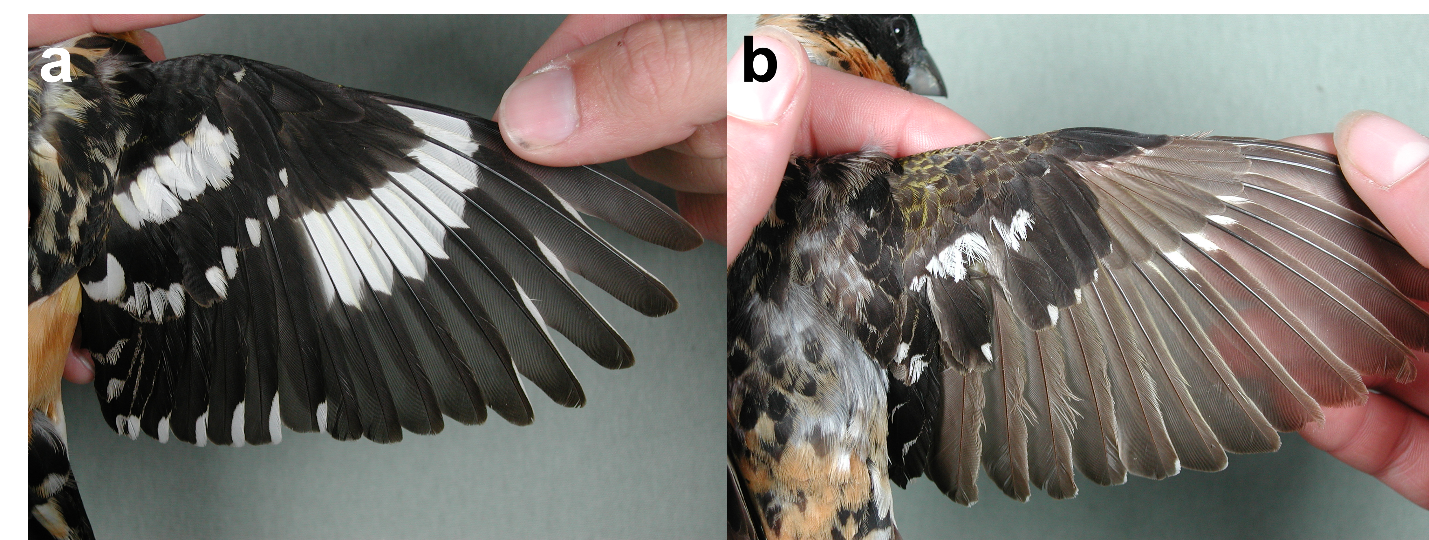


Figure S2. Summary of movement data of four GPS-tagged male black-headed grosbeaks in relation to vegetation greenness (Enhanced Vegetation Index; EVI). The two top panels represent two older birds (9yo and 12+yo both from a single site in Yosemite National Park); the two bottom panels are yearling birds entering their second non-breeding season (one from a second Yosemite site, the other from a site in Golden Gate National Recreation Area in coastal California. Warm colors represent molting season, blue represents winter season. Vertical bands span the time window within which each individual was known to be present in molting or wintering regions and changes in color represent relatively large-scale movements. Movements > 1 km are shown with arrows and labeled with distances. Lines represent mean EVI values for molting sites (orange) and winter sites (blue) across the year of tracking. For the bird identified as Yosemite 3 (bottom right), only molting season data were obtained. For both birds in bottom panels, we split out additional colors in the molting season to highlight additional larger scale movements and show that movements tend to be from sites with earlier greening peaks to sites with later peaks or to sites that remain greener throughout the year.


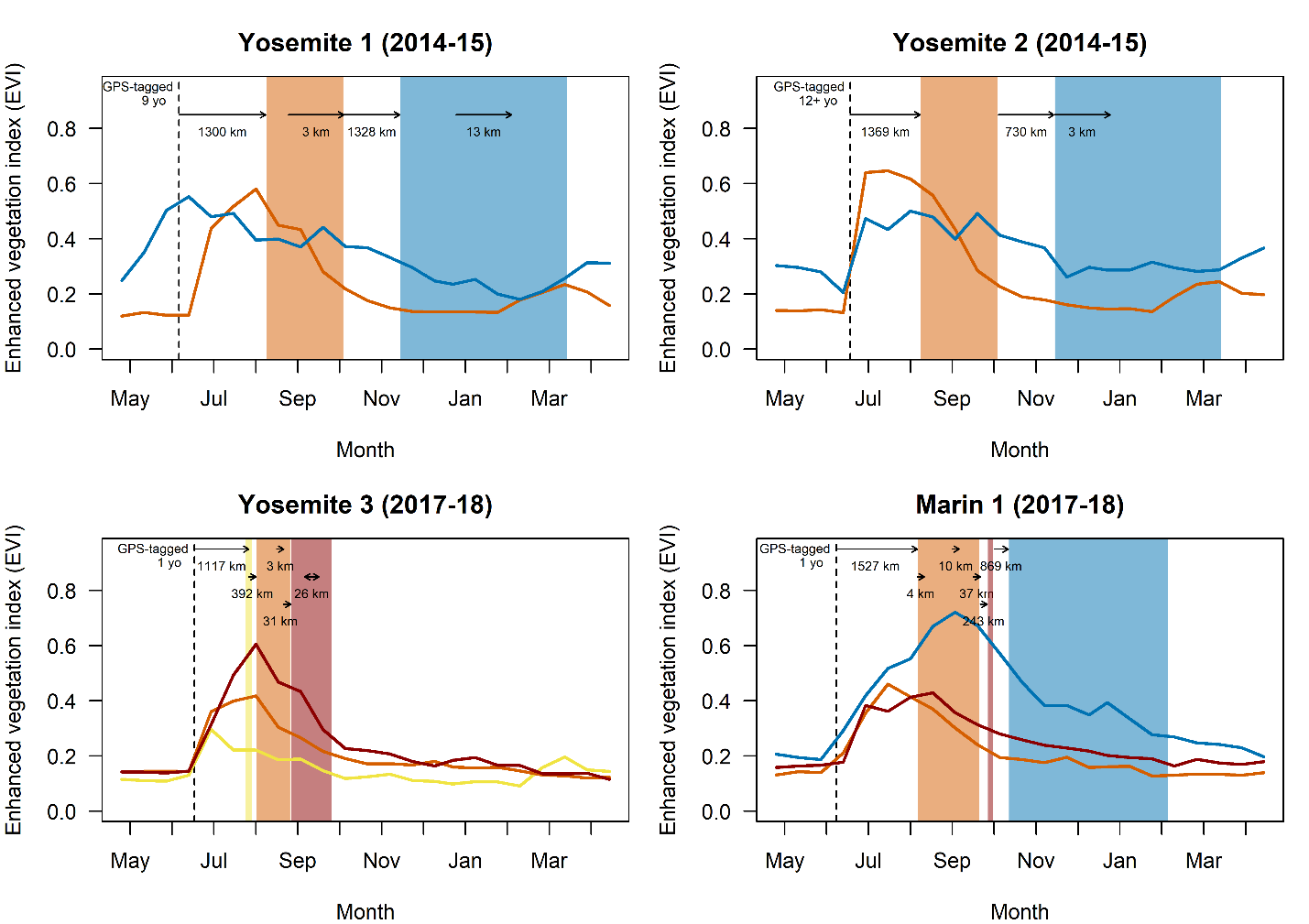

Supplement: Supplementary file 1 — Supplementary Material [file ECE3-12-e8934-s001.doc]
